# DAXX/ATRX and MEN1 genes are strong prognostic markers in pancreatic neuroendocrine tumors

**DOI:** 10.18632/oncotarget.17964

**Published:** 2017-05-18

**Authors:** Joo Kyung Park, Woo Hyun Paik, Kyoungbun Lee, Ji Kon Ryu, Sang Hyub Lee, Yong-Tae Kim

**Affiliations:** ^1^ Division of Gastroenterology, Department of Medicine, Samsung Medical Center, Sungkyunkwan University School of Medicine, Seoul, Korea; ^2^ Department of Internal Medicine and Liver Research Institutute, Seoul National University Hospital, Seoul National University College of Medicine, Seoul, Korea; ^3^ Department of Pathology, Seoul National University Hospital, Seoul National University College of Medicine, Seoul, Korea

**Keywords:** DAXX/ATRX, MEN, neuroendocrine tumor

## Abstract

**Background:**

PanNETs shows heterogeneous biological behaviors. The aim was to investigate prognostic markers based on most frequently mutated genes in PanNETs.

**Results:**

There was a total of 76 patients (M: 39, F: 37) with pathologically proven PanNETs. ATRX/DAXX and MEN1 protein expression was detected in 16 (21%) and 31 (41%) patients, respectively. The mean OS of the total study patients was 16 years, and DFS was 17 years among the 68 patients with curative resections. PanNETs presented with distant metastasis or loss of ATRX/DAXX protein expression was the independent prognostic factors associated with poor OS. In curative resected PanNETs, there was no significant difference in the mean DFS according to ATRX/DAXX or MEN1 protein. However, there was statistically significant difference in survival after the recurrence according to the expression of ATRX/DAXX protein; Y/N: 10 vs. 15 years, *p* < 0.001. In metastatic PanNETs, we could find out OS was significantly longer in negative protein expression of ATRX/DAXX and MEN1 groups; 7 vs. 1 years, *p* < 0.001, 6 vs. 2 years, *p* = 0.02, respectively.

**Materials and Methods:**

The histologically proven PanNETs were enrolled and the clinicopathologic and genetic alterations were evaluated.

**Conclusions:**

Protein expression of MEN1 and DAXX/ATRX can be prognostic markers for PanNETs. Further investigation in genetic alterations of PanNETs may give us insights understanding the behavior of PanNETs.

## INTRODUCTION

Pancreatic neuroendocrine tumors (PanNETs) are rare neoplasms which have an incidence of approximately one per 100,000 individuals per year and represent 3% of all pancreatic tumors [1, 2]. PanNET is an important form of pancreatic neoplasia and the 10-year survival rate is 40% [3, 4]. Several studies have documented a trend towards the increasing incidence and prevalence of PanNETs [5]. Although usually indolent, the biological behavior of PanNET ranges varies from benign to malignant ones, and some PanNETs may also show very aggressive behavior with rapid progression and poor prognosis [6–11]. Predictors of prognosis include non-functioning tumor, tumor size, the presence and the site of metastases, the degree of tumor differentiation, Ki-67 and spontaneous tumor growth rapidity [12–16]. However, the prognostic factors of PanNET are still debatable because of their rarity and heterogeneity of biologic and clinical features.

Recently, *Jiao et al*. and *Marinoni et al*. both conducted the large-scale mutational analysis with the help of high throughput techniques in patients with PanNETs so far. First of all, *Jiao et al*. reported that the gene mutations in MEN1 and DAXX (death-domain-associated protein)/ATRX (α thalassemia/mental retardation syndrome X-linked) genes emerged as the most frequent molecular events and associated with better prognosis [17]. On the other hands, *Marinoni et al*. reported that the loss of DAXX and ATRX are related with chromosome instability and poor survival of patients with PanNETs which seemed to contradictory to the results of Jiao et al. study [17, 18]. The aims of this study were to identify the genetic alterations which enable us to predict the prognosis and further more survival of the patients with PanNETs.

## RESULTS

### Characteristics of the study patients

The baseline characteristics were described in Table 1. The total of 76 patients with pathologically proven PanNETs was enrolled in tertiary, teaching hospital. There were 39 males (51%) and the median age of study patients was 54 years old (range 21 to 76 years). There were 71 patients (93%) who underwent operation including palliative surgery and 68 patients (96%) had curative resections; 51 patients in stage IA,B, 16 in IIA, B and 1 patient who had resection for pancreas mass and single solitary metastasis resection from the liver. Among the resected PanNETs, there were 4 functioning tumors; 2 insulinomas, 1 gastrinoma, and 1 somatostatinoma. The median size of tumor was 23mm ranged from 3 to 200 mm and the followings is the location of PanNETs; head & uncinated: 40, body & tail: 34 and multifocal: 2. In addition, there were 9 (12%) patients who were presented with metastatic PanNETs; AJCC stage IA, IB: 51, IIA, IIB: 16, III:0, IV 9 patients. Among the 68 resected PanNETs, 7 (10%) patients had recurrences after the curative resection.

**Table 1:** Clinicopathologic characteristics

Terms	*n* = 76
Age (median, range, yrs)	54 (21–76)
Sex (M:F)	39: 37
Follow-up (median, range, yrs)	5.9 (1–18.8)
Operation (%)	71 (93%)
Curative resection	68
Palliative operation	3
Functioning tumors (%)	4 (5%)
Size of tumors (median, range, mm)	23 (3–200)
Location	
Head, uncinate process	40 (53%)
Body, tail	34 (45%)
Multifocal	2 (3%)
AJCC Stage	
IA, IB	51 (67%)
IIA, IIB	16 (21%)
III	0
IV	9 (12%)
Recurrence after curative resection	7 (10%)
Grade	
G1	53 (70%)
G2	20 (26%)
G3	3 (4%)
ATRX protein expression (−)	55 (72%)
DAXX protein expression (−)	54 (71%)
MEN1 protein expression (−)	45 (59%)

### Pathologic characteristics

**Figure 1:**
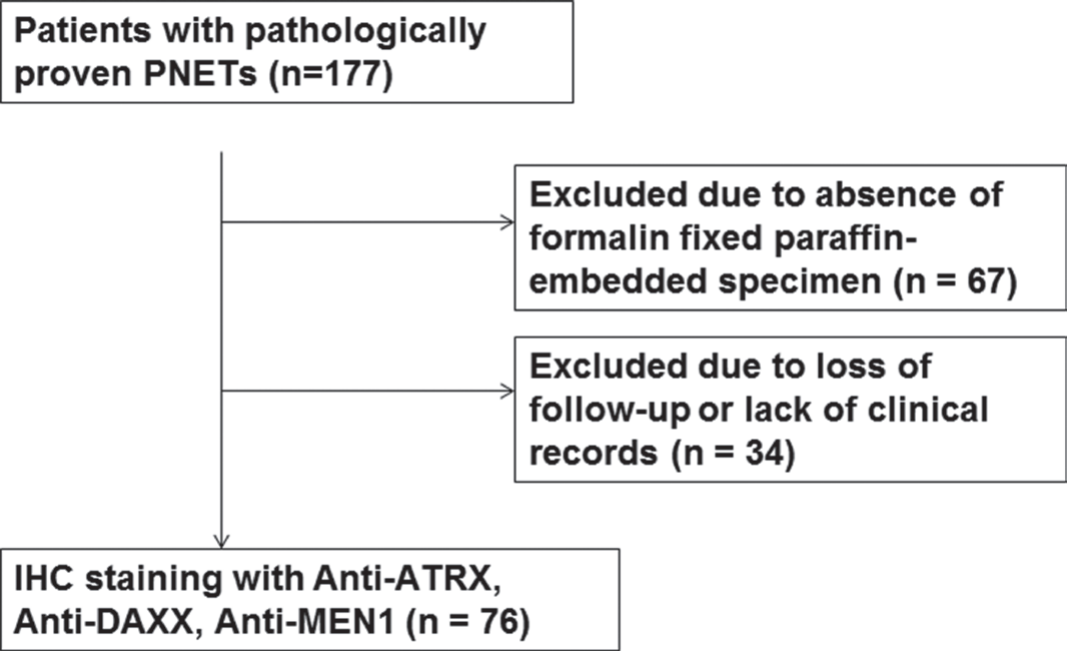
Immunohistochemical staining of MEN1 (A)(D)(G), ATRX (B)(E)(H), and DAXX (C)(F)(I) (**A**–**C**) Positive nuclear staining of MEN1, ATRX, and DAXX (**D**–**F**) Positive cytoplasmic staining of MEN1, ATRX, and DAXX (**G**–**I**) Negative staining of MEN1, ATRX, and DAXX (all pictures x200) (**J**) Expression status of MEN1 and ATRX/DAXX according to the immunohistochemical staining.

All study patients were pathologically proven as PanNETs and the grading system from 2010 WHO classification of neuroendocrine tumors was used for the study patients; G1: 53 (70%), G2: 20 (26%) and G3: 3 (4%). IHC staining results with ATRX/DAXX and MEN1 are shown in Figure 1 and Table 1. In Figure 1, panel A–C showed the positive IHC staining result of DAXX/ATRX and MEN1 which indicated positive expression for DAXX/ATRX and MEN1 proteins. Panel D-I showed negative staining results due to the loss of ATRX/DAXX and MEN1 protein expression. Among the study patients, positive expression for ATRX/DAXX and MEN1 protein were detected in 16 (21%) and 31 (41%) patients, respectively. There were 41 (54%) patients who had both negative expressions for ATRX/DAXX and MEN1 proteins.

### Overall survival and disease-free survival of the study patients

**Figure 2:**
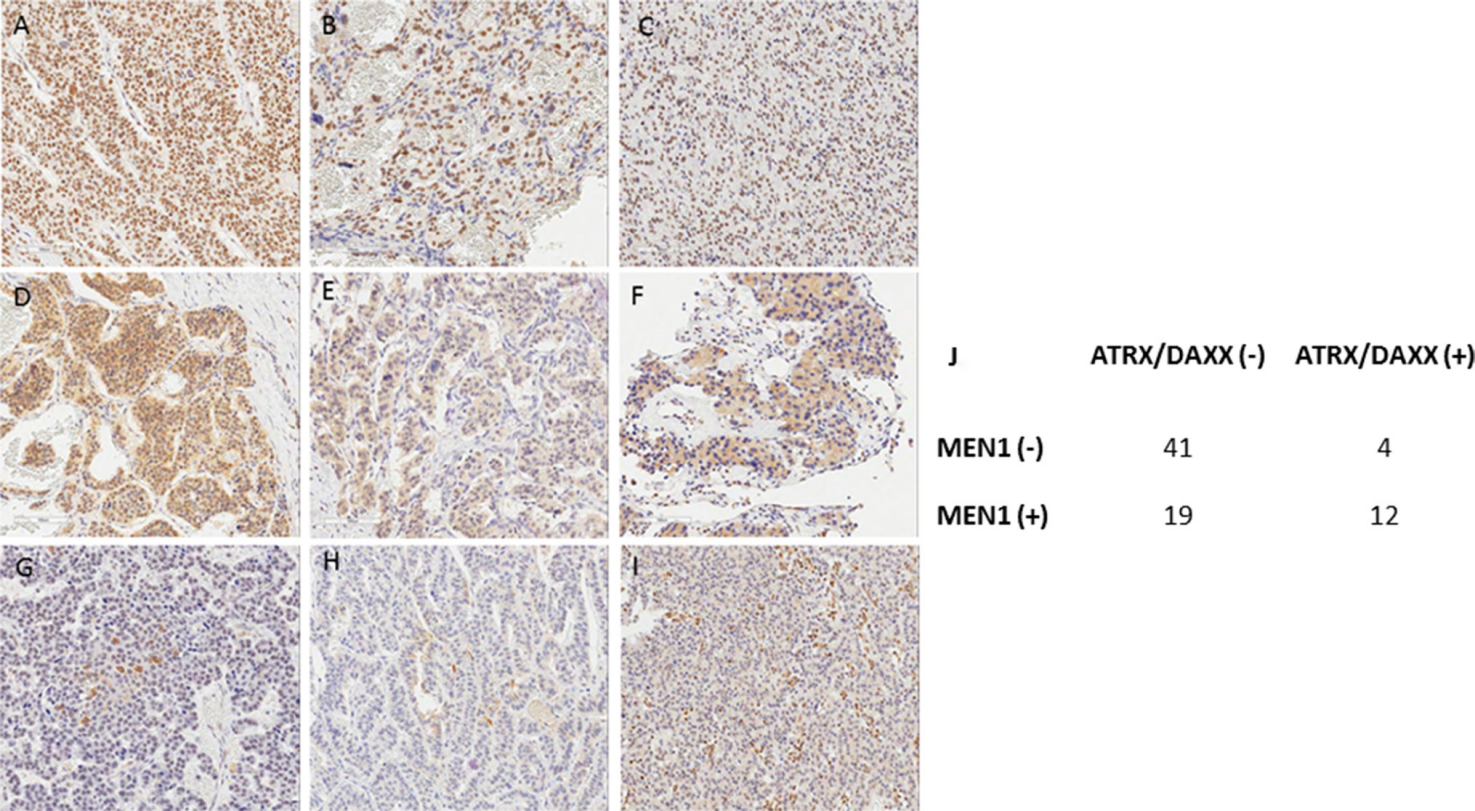
Kaplan-Meier curve of overall survival and DFS of the study patients (**A**) OS of the total of 76 study patients (**B**) DFS of the patients who underwent curative resection.

As we mentioned above, there was a total of 76 study patients and 68 patients who underwent curative resection among them. The mean overall survival (OS) was 15.5 years (95 % CI 13.8–17.1 years) and 16 out of 76 study patients (21%) died during the follow-up (Figure 2A). In addition, DFS was 16.5 years (ranged from 14.9 to 18.1years) among the 68 patients with the curative resections (Figure 2B).

### Prognostic factors affecting OS of the study patients

**Figure 3:**
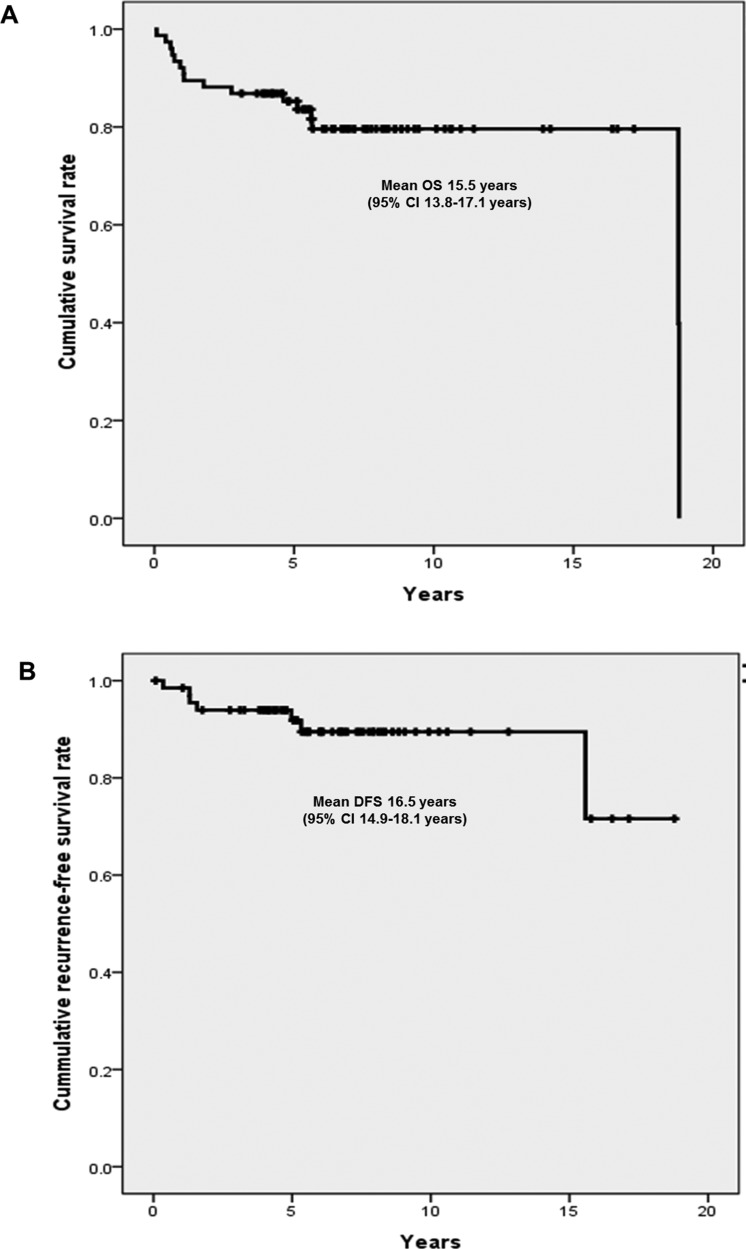
Univariate analyses of OS according to the clinicopathologic factors (**A**) No distant metastasis vs. metastatic PanNETs at the time of diagnosis (mean OS 3.0 yrs vs. 16.9 yrs, *p* < 0.001) (**B**) Lymph node positive vs. negative at the time of diagnosis (mean OS 8.8 yrs vs. 16.0 yrs, *p* = 0.025) (**C**) Whether curative intent surgery was done or not (mean OS 16.7 yrs vs. 2.9 yrs, *p* < 0.001) (**D**) WHO grade (mean OS G1 16.4 yrs vs. G2 15.4 yrs vs. G3 0.6 yrs, *p* < 0.001) and (**E**) Negative for ATRX/DAXX protein expression (N/Y: mean OS 15.3 yrs vs. 10.8 yrs, *p* < 0.001) were statistically significant factors associated with OS among the study patients.

The following clinical and histological parameters associated with OS were analyzed: age, staging at the time of diagnosis, lymph node status, curative intent surgery, WHO grade and protein expression status of ATRX/DAXX and MEN1. Among them, distant metastasis (mean OS 3.0 years vs. 16.9 years, *p* < 0.001), lymph node positive (mean OS 8.8 years vs. 16.0 years *p* = 0.025), curative intent surgery (mean OS 16.7 years vs. 2.9 years, *p* < 0.001), WHO grade (mean OS G1 16.4 years vs. G2 15.4 years vs. G3 0.6 years, *p* < 0.001), and negative expression for ATRX/DAXX protein (mean OS 15.3 years vs. 10.8 years, *p* < 0.001) were significant prognostic factors associated with OS in univariate analysis (Figure 3A–3E). On the other hands, MEN1 protein expression status did not have statistically significant difference in OS (mean OS 16.4 years vs. 14.0 years, *p* = 0.08). In multivariate analysis, patients presented with positive expression of ATRX/DAXX protein (HR 3.809, 95% CI 1.064–13.630, *p* = 0.04) was the only independent prognostic factors associated with poor OS (Table 2).

**Table 2:** Multivariate analysis of prognostic factors affecting overall survival

Variable	HR (95% CI)	*P* value
Lymph node positive	0.482 (0.085–2.722)	0.409
Curative intent surgery	0.412 (0.026–6.605)	0.531
Distant metastasis	7.702 (0.828–71.638)	0.073
WHO grade	1.611 (0.676–3.840)	0.282
**ATRX/DAXX protein expression positive**	3.809 (1.064–13.630)	0.040

### Subgroup analysis in curatively resected PanNETs vs. metastatic PanNETs

**Figure 4:**
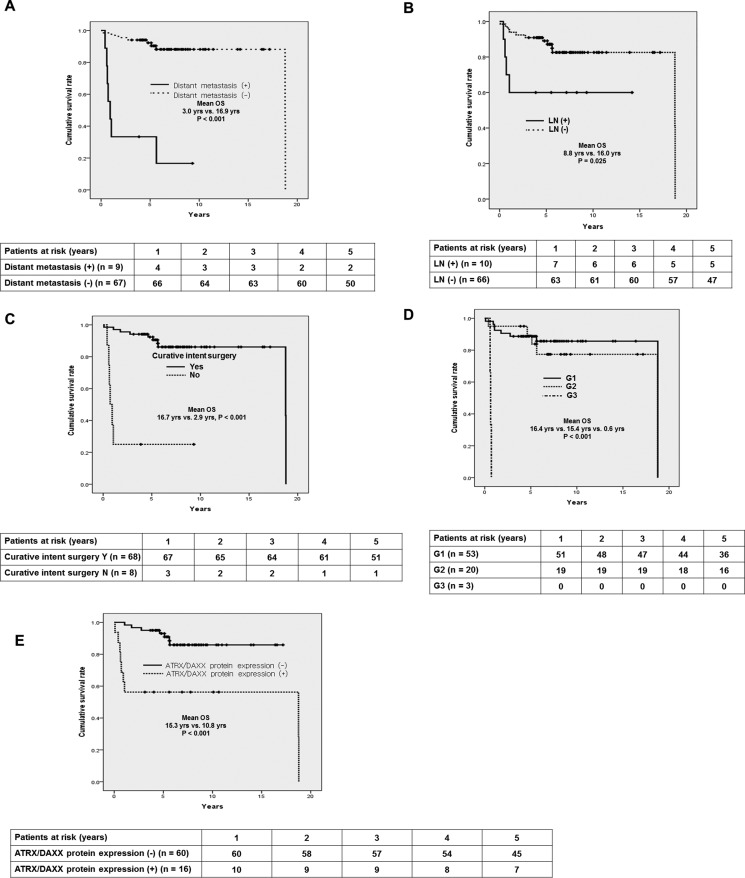
Prognostic factors affecting survival in patients with curatively resected PanNETs (**A**) Kaplan-Meier curves of DFS for ATRX/DAXX protein expression (N/Y: 15.4 yrs vs. 17.1 yrs, *p* = 0.77) and MEN1 protein expression (N/Y: 16.0 yrs vs. 16.4 yrs, *p* = 0.47) (**B**) Kaplan-Meier curves of OS after the recurrence for ATRX/DAXX protein expression (N/Y: 15.3 yrs vs. 9.7 yrs, *p* < 0.001) and MEN1 protein expression (N/Y: 16.4 yrs vs. 12.4 yrs, *p* = 0.08).

There was no significant difference in the mean disease-free survival according to ATRX/DAXX (Y/N: 17.1 years vs. 15.4 years, *p* = 0.77) or MEN1 protein expression status (Y/N: 16.5 years vs. 16.0 years, *p* = 0.47) in curatively resected PanNETs (Figure 4A). However, among the patients with curatively resected PanNETs, both negative protein expression tumors seemed to have longer DFS which was opposite to the result that negative ATRX/DAXX protein expression was the independent prognostic factor for longer OS in the study patients. Therefore, we also evaluated unique parameter such as survival after the recurrence since PanNETs have significantly longer survival compared to PDACs. There was clearly statistically significant difference in survival after the recurrence according to ATRX/DAXX protein expression status; Y/N: 9.7 years vs. 15.3 years, *p* < 0.001 in Figure 4B. On the other hand, MEN1 protein expression status did not make significant difference in survival after the recurrence; Y/N: 12.4 years vs. 16.4 years, *p* = 0.08. However, there was a still tendency to have longer survival after the recurrence in patients with negative MEN1 protein expression.

**Figure 5:**
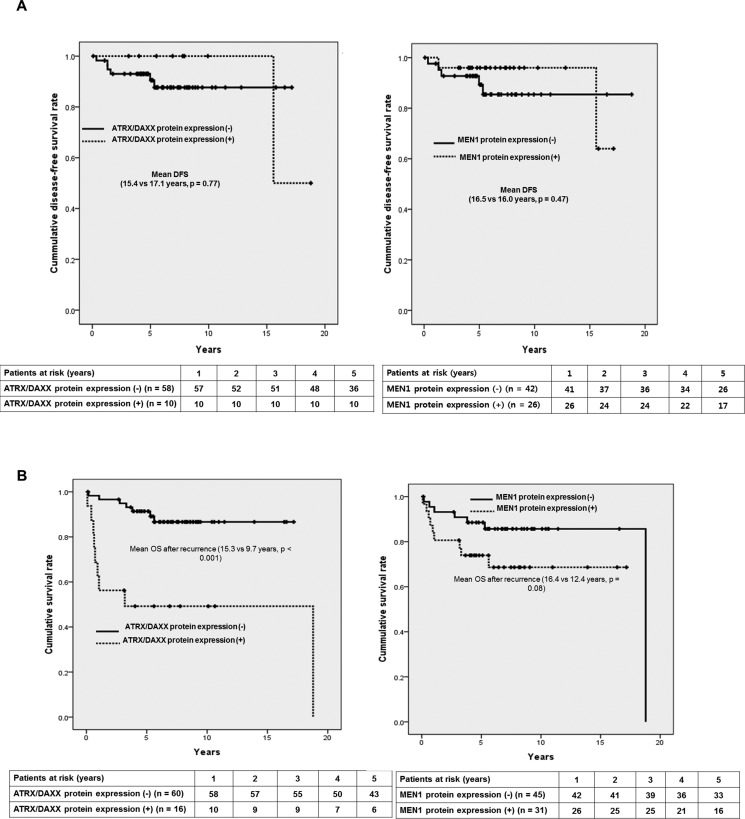
Prognostic factors affecting survival in patients with metastatic PanNETs (**A**) Kaplan-Meier curves of OS according to the protein expression of ATRX/DAXX (N/Y: 6.5 yrs vs. 1.1 years, *p* < 0.001) (**B**) Kaplan-Meier curves of OS according to the protein expression of MEN1 (N/Y: 6.2 yrs vs. 1.5 yrs, *p* = 0.03).

In metastatic PanNETs, we could find out OS was significantly longer in negative ATRX/DAXX and MEN1 protein groups; Figure 5A: 6.5 years vs. 1.1 years, *p* < 0.001, Figure 5B: 6.2 years vs. 1.5 years, *p* = 0.03, respectively.

## DISCUSSION

Hallmarks of PanNETs are having heterogeneous and wide spectrum of clinical paths, however, there is absence of strong prognostic markers for recurrences [19]. The investigation and managements needs to be individualized for each patient and therefore, finding of surrogate markers to predict its prognosis can be very important [20]. The most frequently mutated genes have been reported with the help of high throughput techniques and they are the followings; MEN1 and DAXX/ATRX genes. Somatic mutations of MEN1 and DAXX/ATRX genes were inactivating mutations [17, 21–24]. DAXX/ATRX genes specify proteins implicated in chromatin remodeling. MEN1 gene encodes menin, a histone methyltransferase complex and acts as a tumor suppressor. DAXX/ATRX genes, either of the two subunits consists of a transcription/chromatin remodeling complex [17, 21–24]. To explore and investigate the prognostic markers of PanNETs, we have investigated 76 patients with pathologically proven PanNETs based on genetic alterations and had relatively thorough and longer follow-up data compared to the previous studies [17, 18]. ATRX/DAXX (either one of the genes since they are mutually exclusive) and MEN1 protein expression were detected in 16 (21%) and 31 (41%) patients, respectively. *Jio et al*. have reported that mutation with ATRX/DAXX or ATRX/DAXX and MEN1 have significantly longer OS in metastatic PanNETs [17]. *Marinoni et al*. also collected 149 primary PanNETs from the tumor registry and studied the correlation of loss of DAXX or ATRX expression using IHC staining [18]. The loss of DAXX or ATRX correlated with tumor stage and metastasis, reduced time of relapse-free survival and decreased time of tumor-associated survival [18]. Those two studies have been the relatively large-scale ones compared to previous studies so far and unfortunately, they have suggested different prognostic outcomes according to the ATRX/DAXX mutational status [17, 18]. When we took down the data closely, *Jio et al*. evaluated OS among the 27 metastatic PanNETs and *Marinoni et al*. used two different data sets which had 142 and 101 PanNETs including metastatic patients. However, those two sets of data of *Marinoni et al*. couldn't get the complete survival data; 57/142 (67%) and 37/101 (37%) and metastatic data as well; 104/142 (73%) and 17/101 (17%). In addition, since *Marinoni et al*. study patients were registered from the cancer registries in two different institutions and their follow-ups took place in local general practitioners, and clinical data regarding survival, TNM stage and clinical courses such as recurrence data were not all completely acquired in large number of study patients. Therefore, the total analyzed data became very small number of patients and the important clinical data was incomplete. Unlikely to those previous studies, we have completed all the data set with long-term follow-up and subgroup analyses were done in metastatic and curatively resected PanNETs. First of all, PanNETs presented with distant metastasis (HR 10.124, 95% CI 2.727–37.584, *p* = 0.001) and positive ATRX/DAXX protein expression (HR 4.465, 95% CI 1.382–14.428, *p* = 0.01) were the independent prognostic factors associated with poor OS from multivariate analysis in total study patients. Among the 68 patients with curatively resected PanNETs, ATRX/DAXX or MEN1 protein expression positive tumors seemed to have longer DFS similar to Marinoni study, but it was not statistically significant. Moreover, PanNETs usually have longer survival and heterogeneous clinical courses compared to PDACs (pancreatic ductal adenocarcinomas), we have analyzed unique clinical variable associated with genetic alterations such as survival after the metastasis in curative resected PanNETs. There was statistically significant difference in survival after the recurrence according to ATRX/DAXX protein expression status in curatively resected PanNETs. PanNETs with negative ATRX/DAXX protein expression had significantly longer survival after the recurrences; 15.3 vs. 9.7 years, *p* < 0.001. Also, OS of metastatic PanNETs was significantly longer in negative ATRX/DAXX and MEN1 protein expression group; 6.5 vs. 1.1 years, *p* < 0.001, 6.2 vs. 1.5 years, *p* = 0.03, respectively. Interestingly, metastatic PanNETs and recurred PanNETs after the curative resections have significant different survival according to the mutational status of ATRX/DAXX or MEN1 genes and these result coincided with *Jiao et al*. study. However, we have not done functional studies regarding MEN1 and DAXX/ATRX. It might be also possible that ATRX/DAXX mutually exclusive genes and MEN1 gene mutations would cause silencing the protein expression, and they have been reported to affect the prognosis of PanNETs. The possible mechanisms of MEN1 and DAXX/ATRX in pathogenesis or progression have been reported as follows. The protein product of MEN1, menin, is an substantial component of the MLL/SET1-like histone methyltransferase complex and regulates chromatin remodeling, functioning as activator or suppressor of gene transcription according to the cell type [25]. For example, menin acts as a tumor activator in promoting MLL-dependent leukemias, but acts as a tumor suppressor in neuroendocrine tumors [25]. As we have showed in Figure 4A, patients with positive for MEN1 protein expression had significantly longer DFS compared to negative for MEN1 protein expression group. DAXX mutation decreases p53 levels, diminishing the check point for cellular/DNA damages [25]. In addition, changes in the nucleotide sequence often resulted in nonsense mutations that are generally relevant to tumor suppressor genes [25]. Furthermore, Heaphy et al. reported a perfect correlation between the loss of ATRX or DAXX function and the presence of a telomerase-independent telomere maintenance mechanism known as alternative lengthening of telomeres (ALT) [26]. The association of ATRX and DAXX inactivation with the ALT phenotype might explain previous observations in other tumor types that connected the ALT phenotype with improved prognosis [27, 28]. Thompson et al. also reported that the resulting ALT phenotype is the basis for the good prognosis; probably by preventing the initiation of widespread chromosomal instability [29]. Jiao et al. reported that PanNETs patients with altered ATRX or DAXX genes showed better prognosis than those with wild-type tumors [17]. In Figure 3E, we have also showed that the patients without expression of ATRX/DAXX protein (presumably altered ATRX or DAXX genes) had significantly longer OS compared to positive for ATRX/DAXX protein group.

Understanding of the molecular mechanisms leading to the development of PanNETs can be invaluable for a more personalized treatment approach. Here, we have studied whole sets of PanNETs with different clinical presentations; curatively resected, metastatic and recurrences after the curative resections. ATRX or DAXX loss was proved to be an independent predictor for OS of PanNETs in a multivariate Cox regression analysis including well-established risk factors; tumor stage and tumor grade. Further investigation in genetic alterations of PanNETs may not only give us insights to discover strong prognostic markers for survival but also to predict the behavior of PanNETs.

## MATERIALS AND METHODS

### Study patients

**Figure 6:**
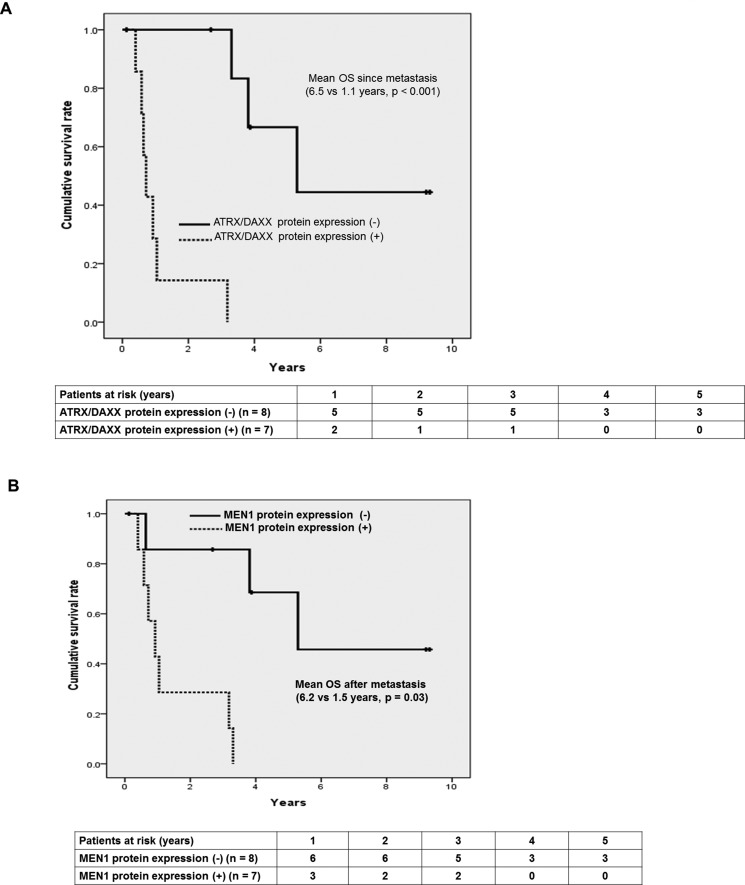
Flow chart of study patients

The total of 177 patients who had pathologically proven PanNETs between January 1990 and March 2012 at Seoul National University Hospital was recruited to this study. The study was reviewed and approved by the institutional review board. Formalin-fixed paraffin-embedded specimens of PanNETs could be collected from the 110 patients. Among them, 34 patients were excluded due to loss of follow-up or lack of clinical records. Finally, 76 patients were included in this study (Figure 6).

### Construction of tissue microarray and immunohistochemical staining

Core tissue biopsy specimens (diameter 2 mm) were obtained from individual paraffin-embedded PanNETs (donor block) and arranged in new recipient paraffin blocks (tissue array blocks) using a trephine apparatus (Superbiochips Laboratories, Seoul, Korea). Each tissue array block contained up to 50 cores, and three array blocks were prepared. An adequate sample was defined as tumor occupying more than 10% of the core area. Immunohistochemical staining was automatically performed by Leica Bond-max autostainer using Bond polymer Refine Detection kit (Leica, Wetzlar, Germany). Four-μm thick glass slides were deparaffinized, dewaxed and hydrated by drying and serial alcohol washing. Primary antibodies were reacted for 15 minutes after heat pretreatment for antigen retrieval (Epitomic retrieval solution, 100°C, 20 minutes) and peroxidase blocking. After post primary and polymer reaction for 8 minutes, chromogenic staining was performed by DAB(3,3-diaminobenzidine) and counter staining by hematoxylin for 1 minute. Detailed information of primary antibody was as follows: 1) MENIN (rabbit polyclonal antibody, 1:200 dilution, Cat# 1397–1, Epitomics); 2) ATRX (rabbit polyclonal antibody, 1:700 dilution, Cat# HPA001906, Sigma); and 3) DAXX (rabbit polyclonal, 1:200 dilution, Cat# HPA008736, Sigma).

### Immunohistochemical staining and pathological interpretation

All three markers were positively stained in cytoplasm, nuclei, or both cytoplasm and nuclei in tumor cells. Considering the normal biologic role of three proteins in nuclei and cytoplasmic interaction with other molecules, staining pattern was assessed by 3 categories, 1) nuclear staining (Nu), 2) cytoplasm only (Cy), 3) negative (N). Criteria of each category was 1) Nu, unequivocal moderate staining in > 5% of nuclei of tumor cells with or without cytoplasmic staining; 2) Cy, unequivocal moderate staining in > 5% of cytoplasm of tumor cells without positive stain in nuclei; 3) N, totally negative staining in cytoplasm and nuclei of tumor cells. The assessment of immunostaining was performed by two pathologists (K.B.L. and M.A.K.). We defined negative expression if the pattern was that of cytoplasmic accumulation with nuclear clearing, as long as adequate internal controls were present. When differences between the observers occurred, the slides were reinvestigated jointly by both investigators and then determined. Representative pictures of nuclear (Nu), cytoplasmic staining (Cy) and negative (N) staining of three markers were displayed in Figure 1. MEN1 was positively stained in nuclei of non-neoplastic acinar cells which were used in internal positive control (Supplementary Figure 1A). ATRX was positively stained in macrophages or lymphocytes which were used in internal positive control (Supplementary Figure 1B). DAXX was positively stained in cytoplasm of non-neoplastic ductal epithelial cells which were used in internal positive control (Supplementary Figure 1C). Positive and negative controls were included with each staining procedure to ensure consistency between consecutive runs.

### Statistical analysis

The overall survival and disease-free survival were analyzed by Kaplan-Meier method, and the significance of differences was determined by the log rank test. Multivariate analysis was performed by Cox proportional hazard regression modeling. A *P* value of < 0.05 was considered to be statistically important. Statistical analysis was conducted by using the IBM SPSS Statistics version 19.0 (SPSS Inc, Chicago, Il., USA).

## SUPPLEMENTARY FIGURE



## Abbreviations

PanNETs: Pancreatic neuroendocrine tumor
IHC: Immunohistochemical staining
PDAC: Pancreatic ductal adenocarcinoma
MEN1: multiple endocrine neoplasia type1
DAXX: death-domain–associated protein
ATRX: a thalassemia/mental retardation syndrome X-linked
OS: Overall survival
DFS: disease-free survival
