# Supplementary material for: DAXX/ATRX and MEN1 genes are strong prognostic markers in pancreatic neuroendocrine tumors
Source: Oncotarget. 2017 May 18;8(30):49796–806. doi: 10.18632/oncotarget.17964 (PMC5564808; doi:10.18632/oncotarget.17964)
Supplement: Supplementary file 1 [file oncotarget-08-49796-s001.pdf]

DAXX/ATRX and MEN1 genes are strong prognostic markers in pancreatic neuroendocrine tumors

Supplementary Materials

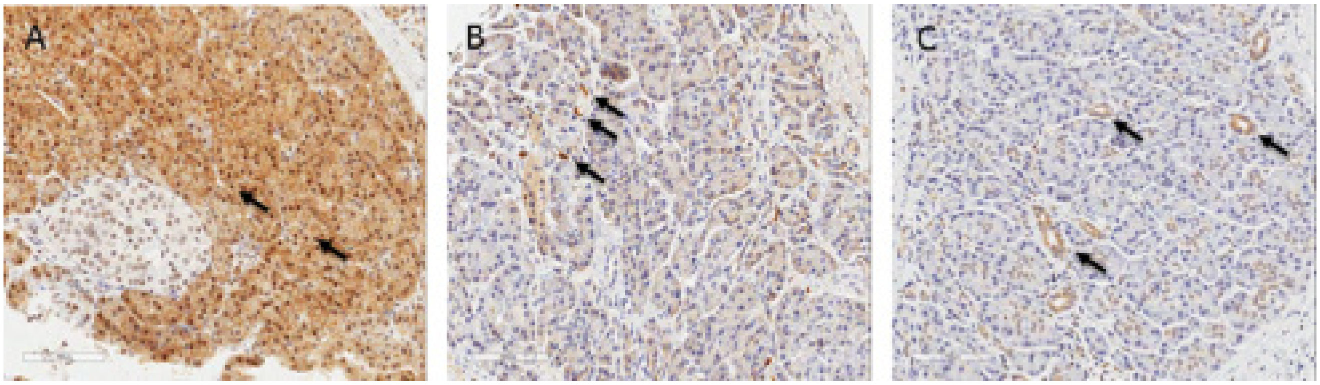

Supplementary Figure 1: Representative pictures of internal control of immunostaining. (**A**) Positive staining of MEN1 in nuclei of non-neoplastic acinar cells MEN1(arrow), (**B**) Positive staining of ATRX in macrophages or lymphocytes (arrow), (**C**) Positive staining of DAXX in cytoplasm of non-neoplastic ductal epithelial cells (all pictures ×200).
